# Overview of Evidence in Prevention and Aetiology of Food Allergy: A Review of Systematic Reviews

**DOI:** 10.3390/ijerph10115781

**Published:** 2013-11-04

**Authors:** Caroline J. Lodge, Katrina J. Allen, Adrian J. Lowe, Shyamali C. Dharmage

**Affiliations:** 1 Centre for MEGA Epidemiology, the University of Melbourne, 207 Bouverie Street, Melbourne, Victoria 2010, Australia; E-Mails: clodge@unimelb.edu.au (C.J.L.); lowe.adrian@gmail.com (A.J.L.); 2 Murdoch Children’s Research Institute, Royal Children’s Hospital, 50 Flemington Road, Parkville, Victoria 3052, Australia; E-Mail: Katrina.Allen@rch.org.au; 3 Department of Allergy and Immunology, Royal Childrens Hospital, 50 Flemington Road, Parkville, Victoria 3052 Australia

**Keywords:** food allergy, aetiology, prevention, overview

## Abstract

The worldwide prevalence of food allergy appears to be increasing. Early life environmental factors are implicated in the aetiology of this global epidemic. The largest burden of disease is in early childhood, where research efforts aimed at prevention have been focused. Evidence synthesis from good quality systematic reviews is needed. We performed an overview of systematic reviews concerning the prevention and aetiology of food allergy, retrieving 14 systematic reviews, which covered three broad topics: formula (hydrolysed or soy) for the prevention of food allergy or food sensitization; maternal and infant diet and dietary supplements for the prevention of food allergy or food sensitization and hygiene hypothesis-related interventions. Using the AMSTAR criteria for assessment of methodological quality, we found five reviews to be of high quality, seven of medium quality and two of low quality. Overall we found no compelling evidence that any of the interventions that had been systematically reviewed were related to the risk of food allergy. Updating of existing reviews, and production of new systematic reviews, are needed in areas where evidence is emerging for interventions and environmental associations. Furthermore, additional primary studies, with greater numbers of participants and objective food allergy definitions are urgently required.

## 1. Introduction

Globally, the prevalence of food allergy appears to be rising. Increases in food allergy incidence have been reported in the UK [1], the USA [2] and Australia [3]. Alarmingly, Australia experienced a 350% increase in hospital admissions for food-related anaphylaxis episodes between 1994 and 2005, mostly in the 0–4 age group [4]. Although the prevalence of food allergy is difficult to measure in the general population, the increasing global prevalence of food related anaphylaxis is likely to reflect an underlying increase in prevalence, which will add substantially to the food allergy health burden. A point prevalence estimate in Australian children, from a unique population-based study using the gold standard of oral food challenge, suggests that the prevalence of infant food allergy might already be as high as 10% in a developed country urbanized setting [5].

Common immunoglobulin E (IgE)-mediated food allergies in early life include: cow’s milk, hen’s egg, and peanut [6]. Most IgE-mediated egg and milk allergies resolve over the first few years [7]. By contrast, peanut allergy, which is more commonly associated with more severe reactions and a higher risk of anaphylaxis [4], is less likely to resolve, with only 20% of infants outgrowing their peanut allergy by the age of 5 years [8]. The apparent increases in infant food allergy prevalence may result in an increase in adulthood food allergy. It has also been proposed that food allergy may be the first step of the allergic march [9] leading to asthma and hay fever. These consequences of food allergy put the increasing health burden into better perspective.

Although there is a known genetic component to food allergy [10], there are several clues that environmental factors may be responsible for the current epidemic. Recent reviews including meta-analysis [6] and systematic reviews [11,12] have found the prevalence of food allergy to be geographically heterogeneous, with estimates ranging from 1–10%. Heterogeneity was found for both self-reported food allergy and food allergy measured objectively using oral food challenges. Other authors have found differences in prevalence within countries according to latitude [13] or remoteness [14]. Geographic heterogeneity of prevalence reflects the findings for other IgE related diseases such as asthma, eczema and hay fever as reported in the International Study of Asthma and Allergies in Children [15] and indicates that the factors driving the increase in food allergy are likely to be environmental. Supporting this hypothesis, studies in migrants have shown that the prevalence of food allergy in individuals is determined by their place of residence [16].

Since it is known that food allergy is most prevalent in infancy and early life, it is important for preventive strategies to focus on environmental exposures which can act pre-natally and/or in the first few years of life. The current theories concerning environmental factors and food allergy are focused on three broad areas:

(1) The direct effect of allergens introduced into infant and maternal diets at specific times and principally whether earlier introduction promotes the development of tolerance in a maturing immune system or the development of allergy;
(2) The role of environmental microbiota in the normal education of the immune system, whereby it is thought that the normal immune system requires the presence of a diverse microbiota in early life for the development of tolerance and a western lifestyle limits exposure to these necessary microbiota (the hygiene hypothesis);
(3) Other lifestyle factors including the effects of vitamin D and environmental pollution on the immune system.

The food allergy epidemic has encouraged the instigation of new primary studies, many of which are still in the recruiting phase. Food allergy is a difficult area to study for several reasons. One of the key issues, as outlined by the 2010 USA guidelines for the diagnosis and management of food allergy [17], and by the International Consensus on Food Allergy [7] is that oral food challenge testing is required to make a firm diagnosis of food allergy. This “gold standard” diagnosis is expensive and time consuming requiring experienced personnel in a controlled setting, with the result that studies tend to be limited to small numbers of participants. Additionally, there is no internationally recognized standard for performing oral food challenges, so even amongst studies utilizing this methodology, there may be measurement heterogeneity. Where resources are not available to perform food challenges, other, less objective, measures of food allergy have been used including: parental report of gastrointestinal and skin symptoms; a doctor’s clinical diagnosis; elevated specific sensitization to a particular food using serum IgE or Skin Prick Testing (SPT) or; a combination of these. It has been shown that in those with reported food allergy, less than 20% have challenge proven food allergy [18], whilst among those with specific food sensitization, less than 50% have challenge proven allergy [19]. Thus, evidence of true food allergy is very poor when these outcomes are used. Another methodological issue is that blinding is not possible for some factors potentially related to food allergy including breast feeding and early solid introduction. Additionally, other factors may lead to participants not contributing information to their assigned exposure groups (*i.e.*, prolonged breastfeeding may delay the introduction of formula), thereby reducing the power of the study.

Given the increasing global disease burden, knowledge concerning the aetiology, prevention and management of food allergy is critical to inform guidelines. Overviews of systematic reviews, which aim to systematically review systematic reviews, are a new approach to synthesize evidence [20], and can be used to inform guidelines. The quality of the information provided by overviews is dependant both on the individual studies included in the systematic reviews and also on the methodological quality of the systematic reviews [21].

We aimed to perform an overview of the food allergy literature, by systematically reviewing all published systematic reviews relating to causation and prevention of food allergy. Synthesizing this evidence will allow the current best evidence to be considered by advisory boards, peak expert bodies and clinicians and translated into best evidence based practice.

## 2. Experimental Section

### 2.1. Eligibility Criteria

We included systematic reviews that addressed primary prevention of or early life associations with food allergy in human children. A systematic review was defined as a review of the literature with a predetermined and transparent search strategy where the search strategy and inclusion and exclusion criteria were explicitly described.

Inclusion criteria: We included systematic reviews of observational and interventional studies in both high risk and population based children.

Exclusion criteria: We excluded studies which were not systematic reviews, and those in adult populations. Additionally we excluded studies where the definition of food allergy was not explicit. We restricted the included articles to English language papers.

### 2.2. Search Strategy

On 14 June 2013 we searched the following databases: PUBMED, EMBASE, The Cochrane Database of Systematic Reviews, and the Database of Abstracts of Reviews of Effects (DARE). The specific search strategies used for each database are included in the Appendix. The search strategies combined a term for food allergy or food hypersensitivity with a term for the type of journal article published (review or systematic review or meta-analysis or quantitative analysis or overview).

### 2.3. Selection of Reviews

The titles and abstracts of the studies obtained from the above search strategy were screened for inclusion by two independent reviewers (C.L. and A.E.). Where differences in judgment of the eligibility occurred, full texts of papers were assessed by both reviewers. Along with these, all studies identified by both reviewers for inclusion from the screening of titles and abstracts were considered as full texts for final inclusion by the same two independent reviewers. Any differences in judgment identified at this stage were adjudicated by a third independent reviewer (A.L.). All excluded studies were recorded. Additionally, references of included articles were screened for potential inclusion.

### 2.4. Assessment of the Quality of the Systematic Reviews

All selected studies were further assessed for study methodological quality using the validated “A measurement Tool to Assess the Methodological quality of Systematic Reviews” (AMSTAR) [22,23]. AMSTAR was created by combining the assessment criteria from two available systematic reviews assessment instruments [24,25] with three criteria based on methodological advances in the field (language restriction, publication bias, publication status). The resulting 37 criteria were applied to 150 systematic reviews. Subsequent factor analysis and review by 11 experts reduced the criteria list to 11 “essential” items [26]. AMSTAR has been validated [22] and is recognized as a preferred tool when performing overviews [27]. Two reviewers (C.L. & A.E.) independently rated study quality using the 11 item AMSTAR checklist, scored as 0 or 1 for each item.

Where differences were noted, these were resolved by discussion between the two reviewers, and where agreement could not be reached, recourse to a third reviewer (A.L.) whose decision was considered final.

One reviewer (A.E.) extracted information from each study into the table of study characteristics, which was verified by a second reviewer (C.L.). Where information concerning included studies was not clear from the systematic review, we extracted information from the original (source) papers.

### 2.5. Analysis

We grouped reviews by topic and ranked by AMSTAR quality score. We considered the studies to be of low quality if the AMSTAR was <3, medium quality if the AMSTAR score was ≥3 and ≤7 and high quality if the AMSTAR score was 8–11. This is the scale employed by the Canadian Agency for Drugs and Technologies in Health, a Cochrane supported body which “provides decision-makers with the evidence, analysis, advice, and recommendations they require to make informed decisions in health care” [28].

## 3. Results and Discussion

### 3.1. Results

**Figure 1:**
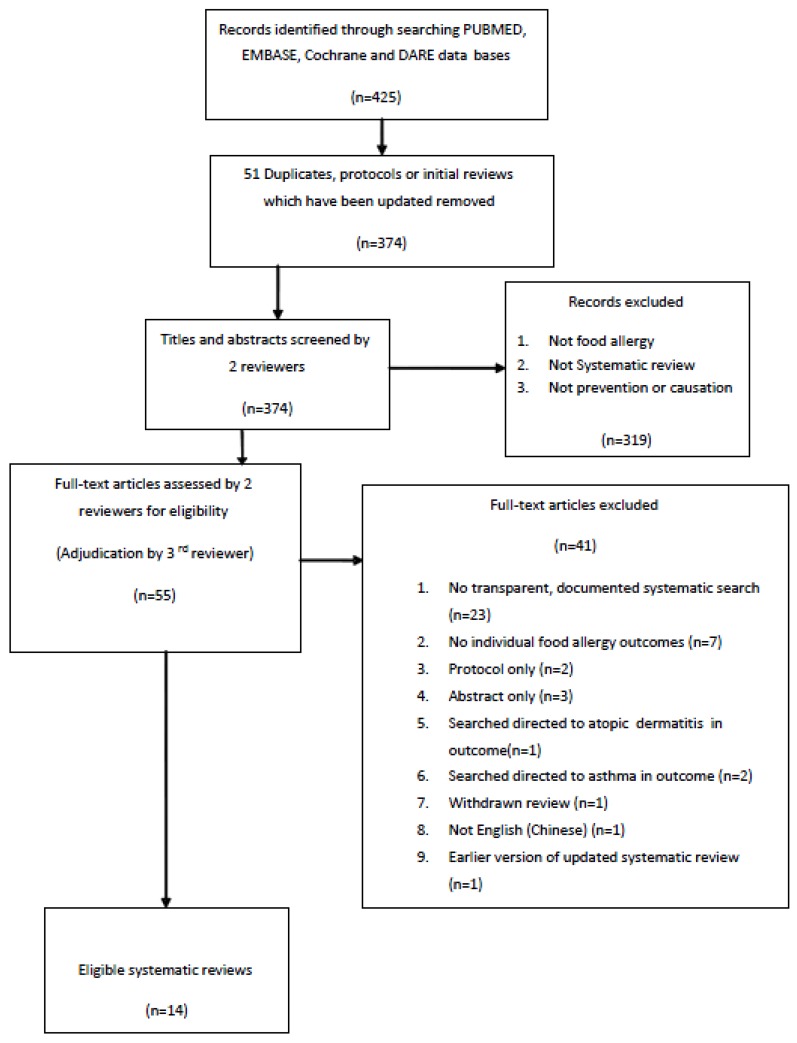
Flow chart of the search process.

The search strategy yielded 425 records and the process is described in Figure 1. After removing duplicates, 374 titles and abstracts were assessed by two reviewers, who identified 55 records for full text assessment. Forty-one articles were subsequently excluded for a variety of reasons including: lack of a systematic search for studies addressing the outcome of food allergy [29,30,31,32,33,34,35,36,37,38,39,40,41,42,43,44,45,46,47,48,49,50,51,52,53,54,55]; no reported individual food allergy outcomes [56,57,58,59,60]; protocol or abstract only [61,62,63,64,65]; withdrawn from the literature [66]; earlier version of updated review [67]; Chinese language [68] and; unable to locate [69].

The remaining 14 systematic reviews fulfilling the search criteria were then rated for quality by two reviewers according to the AMSTAR criteria. The resulting quality assessment scores are given alongside other study parameters in three tables.

We assessed the systematic reviews based on the type of intervention or subject assessed for the prevention of food allergy or food sensitization. Broadly speaking there were three subjects represented by the selected reviews:

(1) Formula (hydrolysed or soy) for the prevention of food allergy or food sensitization.
(2) Maternal and infant diet and dietary supplements for the prevention of food allergy or food sensitization:
  - Introduction of solids or allergenic solids in infants and/or mothers diet.
  - Probiotics and omega-3 supplements to mother and infant.
(3) Hygiene hypothesis related interventions:
  - Infant immunisation and its impact on immune deviation and food allergy/food sensitization development.
  - Delivery by caesarean section.

The following is a description of the review evidence of each of these topics.

#### 3.1.1. Formula for Prevention

There were five systematic reviews (in Table 1) on infant formulas for the prevention of food allergy including two high quality Cochrane reviews [70,71]. All of these assessed effectiveness of formulas in the prevention of food allergy/food sensitization [70,71,72,73,74]. Three reviews [70,73,74] reported results from infants at high risk of food allergy or allergic disease and two reviews [71,72] included both high risk and population based studies.

**Table 1:** Systematic reviews of Infant formulas and the risk of food allergy or food sensitization.

First author (year) AMSTAR quality Meta-analysis (MA)+ or −	Designs of the studies included in each review and search dates	Intervention/s/comparisons	Population/s studied	Outcome/s measured	Main Results	Authors’ Conclusion/s
**Szajewska (2010)** [74] **Quality: High (9)** **MA−**	**1 RCT** 1985–2010	Partially hydrolysed 100% whey formula (pHF) *vs*. Standard infant formula (StF)	**High Allergy risk** Infants with at least 2 first degree relatives with allergy whose mothers decided not to breastfeed	**Food Allergy** (FA) at 6 months. Food Allergy—not defined in SR. Source papers—symptoms suggestive of cow’s milk allergy such as diarrhoea and colic.	RR 0.36, 95% CI (0.15, 0.89) (1 study (n = 67)	Results indicate that pHF is effective in prevention of symptoms of possible CMA compared to StF ☺
**Osborn (2006)** [70] **update (2009)** * Quality: High **(11)** **MA−**	**1 RCT** Search 1980–2006 Included only trials with greater than 80% follow up	Soy Formula *vs*. Cow’s milk formula	**High Allergy risk** Children with biparental history of allergic disease	**Food Allergy** Not defined in SR. Source papers—Not clear—GI symptoms and IgE characterized as Obvious, Probable or possible atopic disease **Cow’s Milk protein intolerance (CMPI) Soy protein allergy** **(SPA)** **Cow’s milk allergy (CMA)**	CMPI RR = 1.09 (0.45, 2.62) SPA RR = 3.26 (0.36, 29.17) CMA RR 1.09 (0.45, 4.86) All results from 1 study (n = 50)	Feeding with soy formula cannot be recommended for primary prevention for infants at high risk of allergy or food intolerance 😐
**Osborn (2006)** * [71] **Quality: High (11)** **MA−**	**5 RCTs** Updated search March 2009 Included only trials with greater than 80% follow up	Hydrolysed infant formula *vs*. human milk or cow’s milk formula AND Partially hydrolysed *vs*. extensively hydrolysed cow’s milk	**High Allergy Risk (3 studies)** 2 studies biparental atopy or uniparental atopy with raised cord IgE 1 study at least 1 first degree relative **Population based (2 studies)** Updated search March 2009	**Food Allergy/CMA1** study unknown 2 studies unblinded food elimination/challenge 2 studies used symptoms with specific IgE	1. Short term studies (2): 1.1 Hydrolysed *vs*. human milk **CMA** RR 7.11, 0.35, 143.84 (n = 90) RR 0.87, 0.52, 1.46 (n = 3,559) **Food Allergy** RR 1.43, 0.38, 5.37 (n = 90) 1.2 Hydrolysed *vs*. cow’s milk **CMA** RR 5.13, 0.25, 103.43 (n = 90) RR 0.62, 0.38, 1.00 (n = 3,559) **Food Allergy** RR 1.37, 0.33, 5.71 (n = 90) 2. Long-term studies (3) 2.1 Hydrolysed vs. cows **CMA** RR 0.36, 0.15, 0.89 (n = 67) **Food Allergy** RR 1.82, 0.64, 5.16(n = 141) 2.2 Extensive *vs*. partial hydrolysed **CMA** RR 0.13, 0.01, 1.16 (n = 246) **Food Allergy** RR 0.43, 0.19, 0.99 (n = 341)	No evidence to support feeding with hydrolysed formula for prevention of allergy compared to breastfeeding. 😐 In high-risk infants unable to be breast fed limited evidence of allergy and CMA reduction. ☺ Need further trials
**Hays (2005)** [72] **Quality: Low (2)** **MA−**	**RCTs** 22 studies	Comparison of hydrolysed formulas with: breastfeeding, cow’s milk formula, soy formula or combinations	**High Allergy Risk** (22) **Population based** (1)	**Atopy** Not defined in SR. Source papers—seems largely based on objective measure in the presence of GI symptoms—open food challenge, DBPCFC, SPT IgE	High-risk infants demonstrate significant reductions in the cumulative incidence of atopic disease through the first 1 to 5 years of life compared with feeding CMF. (no pooled results)	Formulas seem effective but better measures food allergy needed to confirm ☺
**Schoetzau (2001)** [73] **Quality: Medium (5)** **MA−**	**RCTs** **Prospective Cohorts** 3 studies up to 2001 ‡	Comparison of hydrolysed formulas *vs*. cow’s milk formula	**High Allergy Risk**	**Food allergy**: based on strict, well-defined food elimination and challenge procedures including double-blind placebo controlled food challenge.	**Food allergy** 0.50 (0.04; 5.72) (1 study, n = 91) **Sensitization to cow’s milk:** (1 study, n = 91) 9 months 0.19 (0.02; 1.66) 18 Months 0.26 (0.05; 1.32) (1 study, n = 67) 6 months 0.07 (0.00; 1.29) 12 months 0.05 (0.00;1.01)	The lack of statistical power of these studies means that more studies will have to be conducted to determine the effect of hydrolysed formulas and allergy 😐

* Cochrane review; ^‡^ Includes 2, now discredited, studies by Chandra, but results from these studies not used for form these results; ☺ = Intervention associated with prevention of food allergy or food sensitization; 😐 = Intervention not associated with either increased or decreased risk of food allergy or food sensitization.

The most recent three reviews were all of high methodological quality [70,71,74] and concluded that:

- Soy formula could not be recommended for prevention of food allergy/sensitization in high risk children [70].
- There was no evidence to support the use of hydrolysed formulas over breast milk for food allergy/sensitization prevention [71].
- There is insufficient evidence to conclude that the use of hydrolysed formulas may reduce food allergy/sensitization when compared with standard formula in high atopy risk children [71,74].

The assessment of food allergy/sensitization within these reviews varied and was generally poorly defined. Two reviews included studies which used self-reported or physician’s assessment of food allergy based on gastro-intestinal symptoms [71,74]. Two reviews included studies where food allergy was defined either by self-report, physicians report, sensitization or oral food challenge [70,72] and only one review included studies using solely the strict definition of a positive oral food challenge to define food allergy [73]. This review by Schoetzau, *et al*. failed to find an association between the use of hydrolysed formulas and food allergy when compared with the use of cow’s milk formulas, although this result was only based on 351 children from three studies.

The evidence concerning the potential effectiveness of hydrolysed formulas for food allergy prevention comes from reviews by Szajewska, *et al*. [74] and Osborne, *et al*. [71]. Although the review by Szajewska, *et al*. found a reduction in food allergy RR 0.36, 95% CI (0.15, 0.89), this figure was derived solely from one study of 67 infants, exclusively fed formula from birth, in whom the definition of food allergy was based on observation of gastrointestinal symptoms such as diarrhoea and colic, and the effect of withdrawing and reintroducing the food, according to the source text [75]. The Cochrane review by Osborne *et al*. [71] reported only one other study on 3,473 infants [76] where short term feeding in the weeks following birth with hydrolysed formula was associated with a moderate reduction in the risk of cow’s milk allergy (CMA) in infants, when compared with cow’s milk formula (RR 0.62; 95% CI 0.38, 1.00). In this study the diagnosis of CMA was made by oral food challenge. The remaining two, older reviews [72,73] were of lesser methodological quality and both include the now discredited articles by Chandra. Since the publications of these reviews, it was identified that the results from the Chandra articles were likely to have been fabricated [77]. The qualitative review by Hays, *et al*. [72], which was of low quality, also found that the use of hydrolysed formulas may reduce food allergy/sensitization in high allergy risk children when compared with cow’s milk formula.

#### 3.1.2. Maternal and Infant Diet and Dietary Supplements

There were six systematic reviews (Table 2) [67,78,79,80,81,82] which assessed oral exposures to food in mothers and infants and the prevention of food allergy or sensitization. These included two high quality Cochrane reviews [67,80].

**Table 2:** Systematic reviews for maternal and infant diet and dietary supplements and the risk of food allergy/sensitization.

First Author (Year) AMSTAR Quality Meta-analysis MA (+or −)	Study Design/s included in review Search dates	Intervention/s and comparisons	Population/s studied	Outcome measures	Main results	Authors’ Conclusions
**Klemens (2011)** [79] Quality: Medium MA+	**RCT** 3 studies 1950–2010	Omega-3 (n-3 PUFA) supplementation during pregnancy and/or lactation *vs*. placebo (olive or soy oil)	**High Allergy Risk** & **Population based**	**Egg Allergy;** Skin prick test **Food Allergy;** Clinical diagnosis	**Egg SPT** up to 12 months reduced—OR 0.33 (0.16, 0.70) (187 children from 2 studies) **Food Allergy** up to 12 or 30 months—OR 0.46 (0.16, 1.38) (264 children from 3 studies) Supplementation started in pregnancy **Food Allergy** (2 studies on 200 children)—OR 0.34 (95% CI 0.10, 1.15)	n-3 PUFU protective against egg sensitization ☺ but no reduction in food allergy risk 😐
**Anandan (2009)** [78] Quality: Medium MA+	**RCT** 2 studies 1966–2008	Omega-3 (n-3 PUFU) supplementation during pregnancy and/or lactation *vs*. placebo (olive oil)	**High Allergy Risk** & **Population based**	**Food Allergy**—Not defined in SR. Source papers—not clear in one study and clinical diagnosis in other.	**Food Allergy** up to 12 or 30 months RR 0.51 (0.10, 2.55) (148 children from 2 studies)	A non-significant risk reduction in those receiving n-3 PUFU supplements compared to placebo 😐
**Osborn (2007)** * [67] Quality: High MA+	**2 RCT & Quasi RCT** 1966–2007	Probiotics (various types and mixtures) *vs*. no probiotics given to infants	**High Allergy Risk** & **Population based**	**Food Allergy** History of immediate symptoms on food exposure and specific SPT **Cow’s** **Milk** **Allergy** DBPCFC (if suggestive symptoms, signs or SPT)	**Food Allergy** RR 1.54 (0.70, 3.37) (175 children from 1 high risk allergy study using *Lactobacillus acidophilus*) **Cow’s Milk Allergy** RR 0.41 (0.02, 9.84) (72 children from 1 population based study using *Lactobacillus rhamanosus*)	Insufficient evidence to recommend probiotics as a preventative measure for food allergy. 😐
**Kramer (2012)** * [80] Quality: High MA+	**3 RCT & Quasi RCT** 6 July 2012	Maternal dietary antigen avoidance diet (different regimens) during third trimester of pregnancy (2 studies, n = 383) , and pregnancy and lactation (1 study n = 497)	**High Allergy risk**	**Sensitization** Skin prick tests for cow’s milk, egg and peanut allergy at ages 6 months, 1, 2 and 7 years	Many SPTs showed no evidence of association. Those of note: Avoidance during pregnancy: Infant **egg sensitization** at 6 mo RR 0.58 (0.32, 1.05) in 2 studies (n = 340) Avoidance during pregnancy and lactation Child **egg sensitization** at 2 years RR 1.91 (1.03, 3.53) in 1 study (n = 497) Child **milk sensitization** at 2 years RR 4.30 (0.94, 19.67) in 1 study (n = 473)	No significant effect of maternal antigen avoidance on skin prick tests in infant or child 😐
**Thompson (2010)** [82] AMSTAR Quality: Medium MA−	**2 RCTs, ** **2 case controls** 1999–2008	Mother’s exposure to peanut (more or less than once per week) Childs exposure to peanut RCT—Exclusion diets	**High Allergy risk & population based** CC—2 studies of total 48 peanut allergic and 228 controls RCT—2 studies of total 685 full-term newborns	**Sensitization or clinical peanutallergy** Peanut-specific skin prick tests and peanut-specific IgE. Also DBPCFC was used to measure peanut allergy	Due to heterogeneous nature and the small number of studies pooling results was not possible, None of the individual results reported by any of the studies showed any significant association between peanut consumption and food allergy or sensitization	Maternal exposure or introduction time of peanuts in a child’s life appears to have no effect on peanut allergy 😐
**Tarini (2006)** [81] AMSTAR Quality: Low MA−	**1 Prospective cohort study** 1966–2005	Exclusive breastfeeding for 6 months (n = 70) *vs*. introduction solids at 3 months(n = 65)	**High Allergy risk**	**Food allergy**—defined as: history of skin rash or heavy vomiting after ingestion of food by 1 year **At 5 years food allergy** was defined as the above plus positive skin prick test	37% of infants fed solids at 3 months of age had a history of food allergy up to the age of 1 compared to 7% who were fed breast milk exclusively (*p* < 0.001) At 5 years no difference between the two groups	Early solid feeding appears to have no association with food allergy 1 year result due to poor definition of food allergy 😐

* Cochrane review; ☺ = Intervention associated with prevention of food allergy or food sensitization; 😐 = Intervention not associated with either increased or decreased risk of food allergy or food sensitization.

- Timing of solids and allergenic solids for prevention and maternal exposure to allergens.
  Three systematic reviews assessed the role of ingested allergenic foods in mothers and children’s diets and all reached similar conclusions [80,81,82]. One review synthesized the information from three Randomized Controlled Trials (RCTS) concerning the influence of maternal diet during pregnancy and lactation on sensitization in infants and children, finding no relationship between the avoidance of allergenic foods in the maternal diet and sensitization in the child [80]. A second review incorporated two RCTs and two case control studies assessing the role of peanut ingestion in mothers and children, finding no increase in the risk of peanut allergy or sensitization associated with either child or maternal peanut intake [82]. The third review included one cohort study of 135 children and found no difference in the risk of food allergy at the age of 5 years (symptoms plus positive SPT) when comparing infants exclusively breast fed for the first 6 months, to breast fed infants who had solids introduced at 3 months [81]. None of these reviews contributed any evidence concerning whether introducing foods under the cover of breast feeding had an impact on sensitization and food allergy.
- Dietary supplements for prevention (omega 3 and pre/probiotics).
  There were two reviews of RCTs for early life oral interventions using omega-3 polyunsaturated fatty acids (3-PUFA), which were of intermediate quality [78,79]. Both these reviews included the same two original studies [83,84] except that the more recent review incorporated a third study [85]. Both reviews found no association of 3-PUFA supplementation with the risk of food allergy defined as a clinical diagnosis. The more recent review [79] however found a reduced risk for egg sensitization in infancy for those supplemented with 3-PUFA (OR 0.33 (95% CI 0.16, 0.70)) from two studies n = 187.
  One high quality systematic review which summarized RCTs on probiotic supplementation [67], found no association between supplementation and the risk of food allergy, defined by symptoms on food exposure with positive specific SPT (1 study, n = 175) or cow’s milk allergy, defined using double blind placebo controlled food challenge (1 study, n = 72). These results however were based on only 247 children from two different studies in which different lactobacillus species were used.

#### 3.1.3. Hygiene Hypothesis Related Interventions

There were three medium quality systematic reviews (in Table 3) [86,87,88] which considered subjects related to the hygiene hypothesis.

**Table 3:** Systematic reviews of hygiene hypothesis related interventions and the risk of food allergy or food sensitization.

First Author (Year) AMSTAR Quality Meta-analysis MA (+ or −)	Study Designs included in review & search dates	Population/s studied and numbers	Intervention/s and comparisons	Outcome measures	Main results	Authors’ Conclusions
**Arnoldussen (2011)** [86] Quality: Medium MA−	**1 Randomized prospective single blind study** **1 Retrospective Cohort study** No search dates stated	**High allergy risk** RCT- BCG = 62 Placebo = 59 Cohort- Atopic hereditary children 216 cases, 358 controls	BCG vaccination	**Food allergy**: Symptoms of allergy (skin reactions, wheezing, vomiting, or diarrhoea) on more than one occasion after ingestion or contact with a particular type of food or allergen (1 study) Symptoms of feeding induced vomiting diarrhoea or abdominal pain (1 study)	Results not pooled because outcomes were judged to be too heterogeneous on clinical grounds Neither study individually showed a significant association with food allergy	No protective effect of BCG vaccination on the development of food allergy 😐
**Bager (2008)** [87] Quality: Medium MA+	**6 Cohort studies** Between 1966 & 1 May 2007	32,565 children aged 0–17 **Populations not defined**	Delivery by C-section	**Food Allergy/Atopy**: Hospital admission for food anaphylaxis or epipen prescription (age 0–6) (1 study) Physician diagnosis (age 8–17) (1 study) Parent or self report to foods or drugs (age 3–17) (1 study) Parent or self report to egg, fish or nuts (age 1–2) (1 study) Raised specific IgE to food (age 1–2) (2 studies)	**Food allergy or Food atopy** OR 1.32 (95% CI 1.12, 1.55) (6 studies (n = 32,565))	C-section may be associated with increases risk of food allergy. ☹ Results may have been affected by publication bias.
**Koplin (2008)** [88] Quality: Medium MA−	**3 Prospective Cohorts 1 Retrospective cohort** Published before July 2007	**Population based (3) and High Allergy risk (1)** 15,121 children	Delivery by C-Section	**Food Allergy:** Symptoms of food allergy **Sensitization:** IgE antigen-specific levels	Results were not pooled due to small number of papers included in study	C-section may result in an increased risk of IgE-mediated sensitization ☹

☹ = Intervention associated with an increased risk of food allergy or food sensitization; 😐 = Intervention not associated with either increased or decreased risk of food allergy or food sensitization.

##### Infant Immunisation

One systematic review synthesized the evidence on the association between BCG vaccination and the risk of food allergy or food sensitization [86]. This review incorporated one randomized study and one cohort study. Although the results were not pooled, both individual original studies showed no association between BCG vaccination and the risk of food allergy or food atopy.

##### Caesarean Section

Two systematic reviews [87,88], both of medium quality, synthesized the evidence on the association between caesarean section (CS) and food allergy/food sensitization risk from cohort studies. Both these reviews included the same four original studies [89,90,91,92]. Additionally, the review by Bager, *et al*. incorporated two extra studies [93,94] found as a result of differences in search strategies. The six studies retrieved by Bager, *et al*. had five different definitions of their variable food allergy/atopy and differed vastly in the age at which this was measured (0–17 years). Meta-analysis of these six studies yielded an odds ratio of 1.32 (95% CI 1.12, 1.55) for the association between CS and the risk of food allergy/atopy. However, there was some evidence of possible publication bias, based on an asymmetrical funnel plot analysis. The three largest studies (n = 13,980; 8,953; 3,464) did not show an association, whereas the three smallest studies did (n = 2,803; 2,500; 865). Additionally these smaller studies measured their food allergy/atopy outcomes at ages 1 and 2, an age when food allergy is likely to be transient, and two out of these three studies used specific IgE to food allergens rather than diagnosed food allergy. In the other systematic review on CS Koplin, *et al*. [88] did not pool their study results, but noted that there was evidence, from two of their included studies, that delivery by CS may be associated with an increased risk of food allergen sensitization.

### 3.2. Discussion

In this overview of systematic reviews concerning the aetiology and prevention of food allergy, we identified 14 eligible publications. The topics covered by these systematic reviews represented three broad areas concerning prevention and possible aetiology of food allergy: infant formulas (partially and extensively hydrolysed, and soy) (five reviews); early life oral exposures and supplementation (six reviews); and hygiene hypothesis related interventions (three reviews). Our key finding was that there is no robust evidence for association between any of the topics currently covered by systematic reviews and the risk of food allergy in children.

#### 3.2.1. Infant Formulas

Overall there was no compelling evidence that the use of partially or extensively hydrolysed formulas reduced the incidence of food allergy. Only one small review [74] found a reduced risk of food allergy in high-risk children randomized to hydrolysed formula compared to standard formula. A larger, high quality review [71], however did not support this finding and also found no evidence to support the use of hydrolysed formulas over breast milk for prevention of food allergy. The results from the Cochrane review on soy formula [70] were based on a single RCT [95] of only 50 infants, and concluded that soy formula could not be recommended for high allergy risk children, although further original evidence is required on this topic.

#### 3.2.2. Diet and Dietary Supplements

Among other early life oral exposures two reviews concerned supplementation with Omega-3 [78,79] as preventive interventions for food allergy, while one addressed the effect of supplementation with probiotics [67] as preventive interventions for food allergy and three reviewed restriction of mothers and infants diet for prevention of food allergy [80,81,82]. The larger, more recent systematic review on Omega-3 supplementation [78], found evidence of reduced sensitization to egg by 12 months of age but no reduction in food allergy. However, it should be noted that the original studies had limited power, and potentially important protective effects cannot be ruled out at this time. Similarly, no reductions in allergy risk were found in the high quality systematic review assessing the effect of probiotics supplementation [67]. Three systematic reviews assessing the role of ingested antigens in mothers and children through restriction diets [80,82] or delay in solid feeding [81] found no evidence of a relationship with food allergy or food sensitization.

#### 3.2.3. Hygiene Hypothesis Related Interventions

Two systematic reviews of caesarean section (CS) on the incidence of food allergy and food atopy [87,88] reached similar but slightly different conclusions. Koplin, *et al*. found that there may be an increased risk of IgE mediated sensitization following delivery by CS, but did not perform a metaanalysis due to only having four included studies whereas Bager, *et al*. pooled results from six studies with varying outcome definitions and found an increased risk of food allergy or food atopy in those who had been delivered by CS. It appears possible that CS may be associated with increased risk of food sensitization, however this result may also be explained according to the authors of these reviews by both publication bias [87] and possibly by failure to control for the reasons requiring and other circumstances associated with CS [88]. Furthermore, this modest association is unlikely to explain much of the current food allergy epidemic. The systematic review concerning BCG vaccination showed no association with food allergy.

Evidence synthesis from good quality systematic reviews is needed to guide prevention and management of the current global epidemic of food allergy. Although there are many reviews concerning food allergy, the vast majority are narrative rather than systematic. Systematic reviews aim to synthesize the available literature in a methodical, objective manner that can be reproduced and is free from selection bias [96]. Conversely, synthesizing the literature in a narrative way without a pre-determined search strategy, inclusive of all potential articles of interest, may give a skewed subjective interpretation of the literature. Furthermore well conducted systematic reviews are able to shed light on important similarities and differences in the included literature and, by combining study outcomes, may have the power to detect an association in cases where individual studies were limited by participant numbers. The quality of a systematic review depends upon both the quality of the included studies along with the methodological integrity of the systematic review [21].

#### 3.2.4. Major Limitations of Included Systematic Reviews

The quality of the studies included in a systematic review is a major determinant of the quality of the resulting information which can be ascertained from that review [21]. In some of the reviews [87], quality assessments were not made, whilst in others, although these assessments were performed, the quality score or rank was not used to interpret the associations found [85,96]. 

We used the AMSTAR criteria to assess the methodological quality of the systematic reviews. Although this tool was not developed by the Cochrane collaboration, it favours Cochrane reviews as it looks for all the essential steps outlined in Cochrane methodology. 

Most of the systematic reviews pertaining to aetiology and prevention of food allergy synthesized evidence from a small number of included studies, most of which, in turn, enrolled only small numbers of participants. Aggregate results from meta-analyses, therefore, were in many cases performed on limited numbers of both participants and studies making it difficult to draw any firm conclusions.

The definition of food allergy was a major limitation for many of the systematic reviews. In some reviews, no definition was included [78] and tracing the source documents also did not reveal a clear food allergy definition [83]. In others, studies encompassing a myriad of definitions [87] including: specific IgE levels; parental report and; prescription for injectable epinephrine, were grouped together to produce a summary measure. A diagnosis of food intolerance is very different from IgE mediated food allergy in terms of severity, prognosis, and total health burden [7]. Most (87%) of children with food intolerance outgrow this condition by the age of 3 years. Even patients with specific sensitization and suggestive GIT or skin symptoms have less than 50% chance of having true food allergy as defined by oral food challenge [19].

The population was not characterised in some reviews [87]. Pooling studies with different populations may lead to potentially erroneous conclusions. The indication for caesarean section is an important population descriptor delineating a fundamentally different group. For example, the association of caesarean section with allergic outcomes in premature infants may be completely different from the association in babies born at full-term for elective reasons.

## 4. Conclusions

Given the presumed increase in IgE mediated food allergy in the recent past, there is much interest in identifying the causes of food allergy and primary preventive strategies. Our overview suggests that there is still scant evidence concerning aetiology and preventive strategies in the areas that have previously been systematically reviewed. This overview of systematic reviews of food allergy has revealed only 14 reviews encompassing three broad areas and six individual topics, none of which appear to be related, on the current evidence, to food allergy outcomes. The reviews are limited by the diversity of food allergy definitions and lack of participant numbers in their included studies. There is a need for both increased numbers of primary studies with recognized, objective definitions of food allergy outcomes, and for further high quality systematic reviews to synthesize the available evidence on other exposures thought to be linked with food allergy. Notable omissions to the systematic review literature which merit future assessment include: the role of vitamin D supplementation in food allergy and; whether early introduction of egg is associated with a reduction in egg allergy. Both of these topics are currently being investigated by primary studies.

## Appendix

### A1. Search Strategy for the Various Databases

The search strategies combined a term for food allergy or food hypersensitivity with a term for the type of journal article published (review or systematic review or meta-analysis or quantative analysis or overview). The Cochrane and DARE databases did not require the addition of a term encompassing review articles.

#### A1.1. Breakdown of Strategy

##### A1.1.1. Food Allergy Terms

Food allergy; food hypersensitivity.

##### A1.1.2. Systematic Review Terms

Research overview; integrative research; quantitative review; quantitative overview; quantitative synthesis; methodologic review; methodologic overview; systematic review; systematic overview; meta-analysis.

### A2. Search Strategy in PubMed

((((((((((((research AND (integrati * OR overview *))))) OR (((quantitative AND (review * OR overview * OR synthes *))))) OR “Meta-Analysis” [Publication Type]) OR (((methodologic * AND (review * OR overview *))))) OR (((systematic * AND (review * OR overview *))))))))) AND ((“food allergy”) OR “Food Hypersensitivity” [Mesh]) Filters: Humans.

### A3. Search Strategy in EMBase

**Search #**	**Search Terms**
8	7 and 6
7	Food hypersensitivity {Including Related Terms}
6	1 or 2 or 3 or 4 or 5
5	meta analysis {Including Related Terms}
4	Research * (integrati * or overview *) {Including Related Terms}
3	quantitative (review * or overview * or synthes *) {Including Related Terms}
2	Methodologic * (review * OR overview *) {Including Related Terms}
1	Systematic * (review * OR overview *) {Including Related Terms}

### A4. Search Strategy in COCHRANE Database

Food hypersensitivity (MESH).

Limited to Cochrane and other reviews.

### A5. Search Strategy in DARE

Food Hypersensitivity or Food Allergy.
